# Baseline severity of sacroiliitis can predict acute inflammatory status of sacroiliac joint in early axial spondyloarthritis of male patients: a cross sectional study

**DOI:** 10.1186/s12891-019-2549-5

**Published:** 2019-04-04

**Authors:** Hong Ki Min, Hyonjoung Cho, Sung-Hwan Park

**Affiliations:** 1 Division of Rheumatology, Department of Internal Medicine, School of Medicine, The Catholic University of Korea, Seoul St. Mary’s Hospital, 222 Banpo-Daero, Seocho-gu, Seoul, 137-040 South Korea; 2 Division of Rheumatology, Department of Internal Medicine, Armed Forces Capital hospital, Armed Forces Medical Command, Seongnam, South Korea

**Keywords:** Early axial spondyloarthritis, SPARCC, ASAS criteria

## Abstract

**Background:**

This study compared clinical, laboratory and radiographic features of axial spondyloarthritis (axSpA) between ankylosing spondylitis (AS) and non-radiographic axial spondyloarthritis (nrAxSpA) of young male patients. Additionally, we sought factors which can predict the baseline inflammatory status of sacroiliac joint (SIJ) in axSpA.

**Methods:**

We retrospectively reviewed the medical records of 322 patients who visited our hospital due to inflammatory back pain, and 159 male patients with axSpA were enrolled. Enrolled patients were divided into two groups, AS group and nrAxSpA group, and medical records, laboratory data, radiologic findings were collected and analyzed.

**Results:**

Alternating buttock pain and CRP elevation were significantly frequent in AS patients than nrAxSpA patients (68.8% vs 41.3%, *P* = 0.001, 63.5% vs 37.1%, *P* = 0.002), and SPondyloArthritis Research Consortium of Canada (SPARCC) score of SIJ was higher in AS patients than nrAxSpA patients (14.0 vs 5.0, *P* < 0.0001). Baseline sacroiliitis severity, psoriasis, and CRP elevation had positive association in univariate and multivariate regression analysis for SIJ inflammatory SPARCC score.

**Conclusion:**

AS patients were more frequently in acute inflammatory state than nrAxSpA patients according to laboratory and MRI finding. Baseline sacroiliitis grade was significantly associated with baseline inflammatory SPARCC score of SIJ. AS patients might need more intense initial treatment to resolve active inflammatory lesion of SIJ and prevent further radiologic progression.

## Background

Axial spondyloarthritis (axSpA) is a type of inflammatory arthritis that includes ankylosing spondylitis (AS), reactive arthritis, psoriatic arthritis, arthritis associated with inflammatory bowel disease, and undifferentiated SpA [1]. Although AS is a prototype disease, modified New York criteria for AS could only diagnose advanced disease because sacroiliitis on plain radiography was mandatory to fulfill the criteria [2]. The Assessment of SpondyloArthritis International Society (ASAS) has established classification criteria to identify patients with early stages of axSpA: the imaging arm of the criteria requires the presence of sacroiliitis on magnetic resonance imaging (MRI) or radiographs in addition to one SpA feature for patients with chronic low back pain with onset at age ≤ 45 years, while the clinical arm requires instead the presence of HLA B27 positivity in addition to two SpA features, and could include earlier disease, non-radiographic axial SpA (nrAxSpA) [3]. The proportion of patients in whom nrAxSpA progresses to AS is relatively small, with one reported as approximately 12% for 2 years and another as 26% for 15 years [4, 5]. In addition, there are some differences between AS and nrAxSpA, including the proportion of sex and inflammatory markers [6, 7]. Due to these different characteristics of nrAxSpA from AS and low progression rate to AS, some studies reported that nrAxSpA and AS might belong to different disease spectra [8], whereas most studies reported that nrAxSpA is a milder or an earlier form of the disease on the same spectrum of AS [9].

Male predominance was observed in AS, whereas nrAxSpA showed a male-to-female ratio of 1:1 [10]. The radiographic spinal structural progression was more severe in male patients with AS [11, 12], whereas the baseline disease activity measured by Bath Ankylosing Spondylitis Disease Activity Index was higher in female patients [13, 14]. The SPondyloArthritis Caught Early cohort demonstrated that male axSpA patients had higher frequency of HLA-B27 positivity and positive finding of imaging, including sacroiliac joint (SIJ) plain radiography and MRI, than female patients with axSpA [15]. These differences between male and female patients with axSpA and a higher proportion of females with nrAxSpA could introduce a bias in the comparison between AS and nrAxSpA. Furthermore, the comparison between AS and nrAxSpA, based only on male sex, at the time of diagnosis has not been performed.

Treatment of axSpA has two goals: the first is to control inflammation to improve pain and stiffness, and the second is to prevent ankylosis of the spine and SIJ to maintain range of motion [16]. Recently Protopopov et al. revealed that progression of sacroiliitis in plain radiography showed significant correlation with worsening of spinal mobitilty [17]. Severe sacroiliitis was recently discovered to be a predictor of new syndesmophyte development in female patients with AS [18]. Bone marrow oedema (BMO) of SIJ is well known inflammatory lesion in SIJ, and BMO can progress to chronic lesion such as fat metaplasia and eventually forms abnormal new bone at axial joints [19]. Furthermore, Bennet et al. reported that severe BMO observed on SIJ MRI with HLA-B27 positivity could predict progression of nrAxSpA to AS, and Wang et al. showed that more patients with nrAxSpA fulfilling the imaging arm criteria progressed to AS than those fulfilling the clinical arm criteria [5, 20]. Therefore, identifying patients with BMO is important in order to identify patients with nrAxSpA who are at higher risk of progressing to AS and to prevent progression from BMO to fat metaplasia for preventing further structural damage in axSpA.

The present study was designed to compare the features of axSpA including MRI finding between AS and nrAxSpA in young male patients. In addition, we aimed to discover baseline variables associated with inflammatory status of SIJ in axSpA.

## Methods

### Patients

We retrospectively reviewed the medical records of patients who initially visited our hospital, a Korean Armed Forces Hospital, for inflammatory back pain between March 2016 and April 2017. The diagnosis was decided by two rheumatologist, Min and Cho. Inclusion criteria were the following: (1) fulfillment of the ASAS classification criteria for axSpA, (2) 18 to 30 years old, (3) initial diagnosis in our hospital, and (4) with initial radiologic findings and laboratory data. All experiments were conducted in accordance with the Declaration of Helsinki (1964). This study was approved by the Institutional Review Board of Armed Forces Medical Command (AFMC-17056-IRB-17-054).

### Radiologic findings

Initial radiologic data included MRI of the SIJ, computed tomography (CT) of the SIJ, and plain radiography of the pelvic joint, lumbar spine, and cervical spine. Active sacroiliitis was defined as high signal intensity on T2-weighted sequences sensitive for water [21]. Inflammatory status of SIJ was measured quantitatively by SPondyloArthritis Research Consortium of Canada (SPARCC) scoring system [22]. Spine radiography was used to score the modified Stoke Ankylosing Spondylitis Spine Score (mSASSS) and identify the presence of syndesmophytes [23]. The grade of sacroiliitis was measured by SIJ CT [24]. All radiologic finding was determined by two rheumatologist, Min and Cho, and the information of patients were blinded. The average score of both readers was used for analysis.

### Demographic, clinical, and laboratory profiles

Patients’ demographic characteristics (e.g., age, sex, and symptom durations) and laboratory data, including HLA-B27, were collected at the time of diagnosis. Items constituting clinical and imaging arm of ASAS classification criteria for axSpA were recorded. Symptom duration was defined as the time from the first axial symptom to diagnosis. C-reactive protein (CRP) level over 0.3 mg/dL was checked as CRP elevation.

### Statistical analysis

Continuous variables are displayed as median and interquartile range. The Mann-Whitney test was used to compare continuous values between groups. Categorical variables, such as proportions, were compared between groups using the chi-squared test or Fisher’s exact test. Inter-reader reliability of mSASSS and SIJ inflammatory SPARCC score were measured by intraclass correlation coefficients (ICC). Factors related with inflammatory SPARCC score of SIJ were identified by performing linear regression analysis. In multivariate regression analysis, factors yielding *P* < 0.05 by univariate analysis and HLA-B27 positivity were included. Values of *P* < 0.05 were considered statistically significant. All tests were performed by R software (R for Windows 3.3.2; The R Foundation for Statistical Computing, Vienna, Austria).

## Results

### Comparison between AS and nrAxSpA in aspect of baseline demographic characteristics, laboratory profiles, clinical features, and radiographic findings

**Fig. 1:**
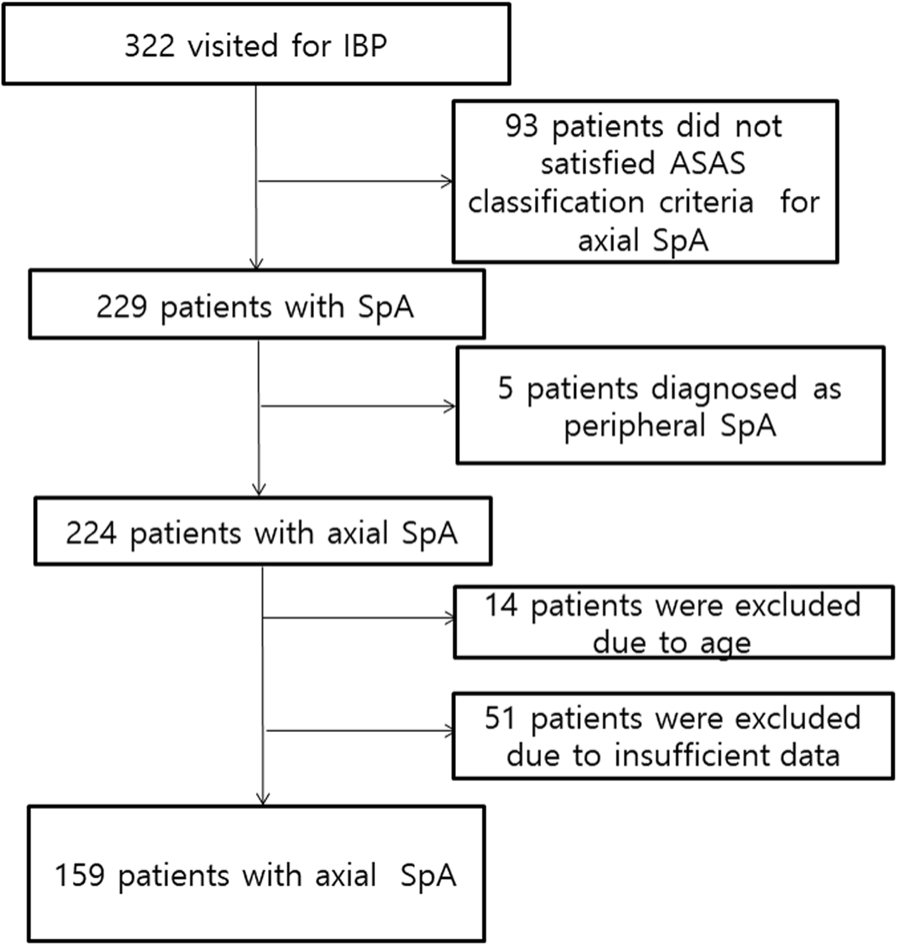
Flow chart showing path for inclusion and exclusion of the patients in the study. ASAS: Assessment of SpondyloArthritis International Society, IBP: inflammatory back pain, SpA: spondyloarthritis

A total of 322 patients visited for chronic inflammatory back pain, and of these 224 patients were diagnosed with axSpA based on ASAS classification criteria [3]. Fourteen patients did not fulfill the age criteria, and 51 patients dropped out due to insufficient data (Fig. 1). We enrolled 159 patients with axSpA, and all patients were men. Demographic, clinical, laboratory, and radiographic findings were compared between AS and nrAxSpA (Table 1). Alternating buttock pain and CRP elevation were more frequently expressed in AS group, whereas enthesitis of heel was more frequent in nrAxSpA group. Inflammatory SPARCC score of SIJ was significantly higher in AS group than nrAxSpA group. Interobserver ICC for mSASSS and inflammatory SPARCC score of SIJ were 0.88 (95% confidence interval [95% CI] 0.83, 0.91) and 0.92 (95% CI 0.90, 0.95), respectively.

**Table 1:** Comparison between AS and nrAxSpA

	nrAxSpA (*N* = 63)	AS (*N* = 96)	*P*
Age (years)	21.0 [20.0;21.0]	21.0 [20.0;22.0]	0.739
Symptom duration (years)	1.0 [0.3; 2.0]	2.0 [0.5; 3.0]	0.186
Peripheral arthritis (%)	36 (57.1%)	41 (42.7%)	0.105
Enthesitis (%)	25 (39.7%)	16 (16.7%)	**0.002**
Uveitis (%)	7 (11.1%)	14 (14.6%)	0.694
Dactylitis (%)	3 (4.8%)	1 (1.0%)	0.343
Psoriasis (%)	1 (1.6%)	3 (3.1%)	0.930
Inflammatory bowel disease (%)	0	0	1.000
CRP elevation (%)	23 (37.1%)	61 (63.5%)	**0.002**
Alternating buttock pain (%)	26 (41.3%)	66 (68.8%)	**0.001**
Good response to NSAID (%)	42 (66.7%)	61 (63.5%)	0.815
Family history of SpA (%)	9 (14.3%)	13 (13.5%)	1.000
HLA-B27 positivity (%)	49 (77.8%)	85 (88.5%)	0.109
ESR (mm/hr)	2.0 [2.0;11.0]	10.5 [3.0;23.0]	**< 0.0001**
CRP (mg/dL)	0.1 [0.0; 0.8]	0.6 [0.2; 1.6]	**< 0.0001**
Right sacroiliitis grade	1.0 [0.0; 1.0]	3.0 [2.0; 3.0]	< 0.0001
Left sacroiliitis grade	1.0 [0.0; 1.5]	3.0 [2.0; 3.0]	< 0.0001
mSASSS	0.0 [0.0; 0.0]	0.0 [0.0; 0.0]	0.088
Presence of syndesmophyte (%)	0 (0.0%)	6 (9.2%)	0.110
SPARCC score of BMO (0–48)	4.0 [0.0; 8.5]	12.0 [7.0;19.0]	< 0.0001
SPARCC score of intense oedema (0–12)	0.0 [0.0; 0.0]	0.0 [0.0; 0.5]	0.015
SPARCC score of deep oedema (0–12)	0.0 [0.0; 0.0]	2.0 [0.0; 5.5]	< 0.0001
Total inflammatory SPARCC score of SIJ (0–72)	5.0 [0.0; 9.5]	14.0 [7.0;25.5]	**< 0.0001**

*AS* ankylosing spondylitis, *BMO* bone marrow oedema, *CRP* C-reactive protein, *ESR* erythrocyte sedimentation rate, *HLA* human leukocyte antigen, *mSASSS* modified Stoke Ankylosing Spondylitis Spine Score, *nrAxSpA* non-radiographic axial spondyloarthritis, *NSAID* nonsteroidal anti-inflammatory drug, *SPARCC* SPondyloArthritis Research Consortium of Canada

### Factors associated with total inflammatory SPARCC score of SIJ in patients with early male axSpA

**Fig. 2:**
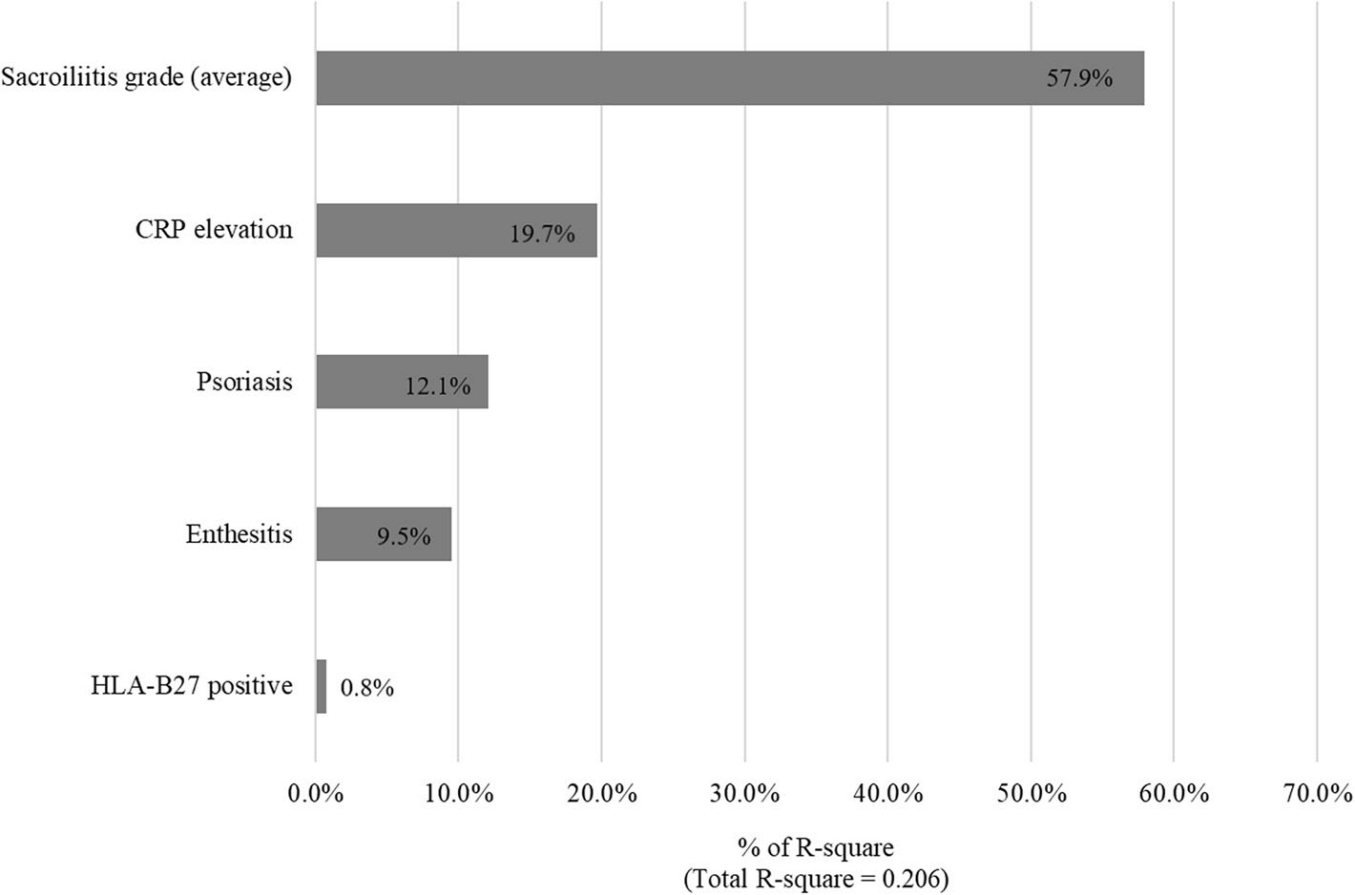
Relative importance of predictive variables on inflammatory SPARCC score of SIJ

Univariate and multivariate linear regression analyses were performed to identify factors associated with total inflammatory SPARCC score of SIJ in patients with early male axSpA. In univariate linear regression analysis, enthesitis (β = − 5.197, 95% CI -9.676, − 0.718) showed negative association, whereas psoriasis (β = 12.976, 95% CI 0.423, 25.529), CRP elevation (β = 6.220, 95% CI 2.332, 10.108), and average grade of sacroiliitis (average of right and left sacroiliitis; β = 4.823, 95% CI 3.076, 6.570) showed positive association with inflammatory SPARCC score of SIJ. Multivariate regression analysis showed that psoriasis (β = 12.339, 95% CI 0.601, 24.078), CRP elevation (β = 3.893, 95% CI 0.113, 7.672), and average grade of sacroiliitis (β = 3.855, 95% CI 1.960, 5.750) were positively associated with inflammatory SPARCC score of SIJ. The aforementioned results are summarized in Table 2. In multivariate regression analysis, the average grade of sacroiliitis was the most important factor of predicting inflammatory SPARCC score of SIJ (Fig. 2).

**Table 2:** Univariate and multivariate linear regression analysis of inflammatory SPARCC score of SIJ

	Univariate analysis			Multivariate		
	β	95% CI	*P*	β	95% CI	*P*
Age (years)	0.055	−0.899, 1.008	0.910			
Symptom duration (years)	0.303	−0.460, 1.065	0.434			
Peripheral arthritis	−1.479	−5.458, 2.499	0.464			
Enthesitis	−5.197	−9.676, −0.718	**0.023**	−2.868	−7.224, 1.489	0.195
Uveitis	−4.286	−10.130, 1.558	0.149			
Dactylitis	4.000	−8.703, 16.703	0.535			
Psoriasis	12.976	0.423, 25.529	**0.043**	12.339	0.601, 24.078	**0.040**
CRP elevation	6.220	2.332, 10.108	**0.002**	3.893	0.113, 7.672	**0.044**
Alternating buttock pain	3.192	−0.810, 7.194	0.117			
Good response to NSAID	2.250	−1.904, 6.405	0.286			
Family history of SpA	−2.965	−8.715, 2.784	0.310			
HLA-B27 positivity	1.781	−3.684, 7.245	0.521	0.038	−5.076, 5.153	0.988
Sacroiliitis grade (average)	4.823	3.076, 6.570	**< 0.001**	3.855	1.960, 5.750	**< 0.001**
mSASSS	0.618	−0.849, 2.085	0.405			
Presence of syndesmophyte	7.419	−2.851, 17.689	0.155

## Discussion

The purpose of our investigation was to compare characteristics between patients with AS and nrAxSpA especially in aspect of inflammatory status of MRI finding, in young male patients. In addition, we discovered baseline variables which had significant association with baseline SIJ inflammatory score of MRI. ASAS classification criteria for axSpA is worthy because it included extra-articular features, such as uveitis, psoriasis in clinical arm, and could diagnose nrAxSpA [3]. Another important point in ASAS classification criteria is that it defined active sacroiliitis on MRI and included MRI finding in imaging arm [3, 21]. Some studies compared features between AS and nrAxSpA, but the patients enrolled in these studies had longer disease duration than the present study [8, 25]. The present study is notable with respect to the study population comprising only male patients, initial information recorded when the diagnosis was made, and baseline MRI findings of the SIJ. Jeong et al. recently compared clinical and laboratory features of AS with nrAxSpA in Korea [25], but it did not include MRI finding and several SpA features, such as family history, nonsteroidal anti-inflammatory drug response, and alternating buttock pain. Significant difference between AS patients and nrAxSpA patients was observed in several aspects. Elevated CRP and alternating buttock pain were significantly more common in AS group, and level of inflammatory biochemical parameters and total inflammatory SPARCC score of SIJ were significantly higher in AS group than nrAxSpA group. The results for the SPondyloArthritis Caught Early cohort showed that inflammatory lesions of the SIJ showed a significant association with buttock pain [26], and this might support the higher frequency of alternating buttock pain and higher inflammatory SPARCC score of SIJ in the AS group. Aforementioned results support that AS patients are more frequently in acute inflammatory state than nrAxSpA in aspect of biochemical parameters and MRI finding. Previous research from a French cohort showed that the baseline radiologic lesions in early axSpA was positively associated with CRP level and active inflammation shown in MRI of the SIJ [27], and these results are consistent with the present study. The aforementioned cohort study has some difference with the present study with respect to the definition of radiologic lesions, which included spinal lesions, and the present study evaluated inflammatory lesion of SIJ by quantitative manner. The results of regression analysis showed significant association between baseline sacroiliitis grade and inflammatory score of SIJ. Sacroiliitis grade was most important factor which could predict the inflammatory SPARCC score of SIJ. Therefore it could support that axSpA patients with severe baseline sacroiliitis have higher probability of possessing active inflammatory lesion in SIJ.

One research showed that high-grade sacroiliitis was associated with development of new syndesmophytes in female patients [18], and another study showed that baseline sacroiliitis fulfilling the modified New York criteria was associated with spinal radiographic progression in univariate analysis [28]. A study from Germany have shown that progression of structural damage in SIJ was associated with worsening of spinal mobility independently with spinal structural damage and disease activity [17]. Active inflammatory lesion on MRI is known to develop chronic lesion such as fat metaplasia, and fat metaplasia has increased risk of new bone formation [29, 30]. Reverting acute inflammatory lesions before these lesions become chronic lesion, fat metaplasia, which is irreversible and have higher risk of abnormal new bone formation, is important. Chen et al. discovered that peripheral blood mononuclear cell from AS patients with severe sacroiliitis had greater ability to express bone morphogenic protein genes by tumor necrosis factor-α and interleukin-1β stimulation than AS patients without severe sacroiliitis [31], which means that severity of sacroiliitis might have correlation with potency of new bone formation in AS. The present study revealed that AS has higher inflammatory status than nrAxSpA, and severity of sacroiliitis in plain radiography was positively associated with inflammatory score of SIJ MRI. Therefore more intense therapy might be needed to AS patients than nrAxSpA patients in purpose to attenuate baseline active inflammatory lesions in axial joints and prevent further radiographic progression.

A previous study from Denmark showed a positive association between HLA-B27 and BMO of SIJ [32]. In the present study, HLA-B27 positivity was not associated with inflammatory SPARCC score of SIJ. This may be attributed to the differences in the study population included in each study. A previous study included patients with chronic low back pain, whereas the present study included only patients with axSpA who fulfilled the ASAS classification criteria for axSpA. In addition, the number of patients with HLA-B27 positivity was relatively high in the present study, and the present study only included male patients (Arnbak B et al. vs the present study: HLA-B27 positivity, 10% vs. 84%; proportion of male patients, 49% vs. 100%) [32]. Further studies with a larger sample size are needed to clarify the association between HLA-B27 and inflammatory SPARCC score of SIJ.

Data from SPondyloArthritis Caught Early cohort showed the BMO of SIJ is not a specific finding for axSpA and could even be present in healthy controls, runners, and women with postpartum back pain [33]. However, the inflammatory SPARCC score of SIJ was higher in the axSpA than in the control group [33]. Furthermore, the factors, such as CRP elevation, associated with the inflammatory SPARCC score for the SIJ, identified in the present study, could aid in distinguishing between the patients with axSpA and controls with SIJ BMO, such as runners and patients with postpartum back pain.

The present study is the first study which compared initial data of AS and nrAxSpA, in young male patients. Although the present study has strength in aforementioned aspect, but several limitations exist. First, because it was a retrospective review, some clinical information such as BASDAI is lacking. However, all patients had precise laboratory data and CT/MRI of the SIJ. In addition, all laboratory and radiologic data were collected from a single tertiary hospital; thereby, data were standardized. It is noteworthy that the sacroiliitis grade was measured by CT in our study. Plain radiography of the pelvis is still the standard method of grading sacroiliitis, but it could be interfered by variable factors, such as bowel gas, whereas CT of the SIJ can exclude such factors and more accurately measure sacroiliitis grade [34]. All patients enrolled in the present study needed CT of the SIJ because the precise sacroiliitis grade decided whether patients could continue their military service. Second, the present study included only male patients with axSpA, therefore results of present study could not apply to female axSpA patients. This originated from the distinctiveness of the Korean army, wherein all young male citizens are conscripted except for few disqualified persons. Third, this study is a cross-sectional study and included only baseline data.

## Conclusions

Several differences were observed between AS group and nrAxSpA group of male patients, especially inflammatory status was higher in AS group than nrAxSpA group. Baseline grade of sacroiliitis, presence of psoriasis, and CRP elevation were significantly associated with inflammatory SPARCC score of SIJ. This could support that early male axSpA patients with severe sacroiliitis might need more prominent therapy to diminish inflammatory lesion of SIJ from the beginning of treatment.

## Abbreviations

AS: Ankylosing spondylitis
ASAS: Assessment of SpondyloArthritis International Society
axSpA: Axial spondyloarthritis
BMO: Bone marrow oedema
CRP: C-reactive protein
CT: Computed tomography
HLA: Human leukocyte antigen
MRI: Magnetic resonance imaging
mSASSS: Modified Stoke Ankylosing Spondylitis Spine Score
nrAxSpA: Non-radiographic axial spondyloarthritis
SIJ: Sacroiliac joint
SPARCC: SPondyloArthritis Research Consortium of Canada
